# Protocol for the Reconstructing Consciousness and Cognition (ReCCognition) Study

**DOI:** 10.3389/fnhum.2017.00284

**Published:** 2017-06-07

**Authors:** Kaitlyn L. Maier, Andrew R. McKinstry-Wu, Ben Julian A. Palanca, Vijay Tarnal, Stefanie Blain-Moraes, Mathias Basner, Michael S. Avidan, George A. Mashour, Max B. Kelz

**Affiliations:** ^1^Department of Pharmacology, Perelman School of Medicine, University of PennsylvaniaPhiladelphia, PA, United States; ^2^Department of Anesthesiology and Critical Care, Perelman School of Medicine, University of PennsylvaniaPhiladelphia, PA, United States; ^3^Department of Anesthesiology, Washington University School of Medicine, Washington University in St. LouisSt. Louis, MO, United States; ^4^Department of Anesthesiology, University of MichiganAnn Arbor, MI, United States; ^5^School of Physical and Occupational Therapy, McGill UniversityMontreal, QC, Canada; ^6^Department of Psychiatry, Perelman School of Medicine, University of PennsylvaniaPhiladelphia, PA, United States; ^7^Center for Sleep and Circadian Neurobiology, Perelman School of Medicine, University of PennsylvaniaPhiladelphia, PA, United States

**Keywords:** emergence, consciousness, cognition, neurobehavioral, sleep, isoflurane anesthesia

## Abstract

Important scientific and clinical questions persist about general anesthesia despite the ubiquitous clinical use of anesthetic drugs in humans since their discovery. For example, it is not known how the brain reconstitutes consciousness and cognition after the profound functional perturbation of the anesthetized state, nor has a specific pattern of functional recovery been characterized. To date, there has been a lack of detailed investigation into rates of recovery and the potential orderly return of attention, sensorimotor function, memory, reasoning and logic, abstract thinking, and processing speed. Moreover, whether such neurobehavioral functions display an invariant sequence of return across individuals is similarly unknown. To address these questions, we designed a study of healthy volunteers undergoing general anesthesia with electroencephalography and serial testing of cognitive functions (NCT01911195). The aims of this study are to characterize the temporal patterns of neurobehavioral recovery over the first several hours following termination of a deep inhaled isoflurane general anesthetic and to identify common patterns of cognitive function recovery. Additionally, we will conduct spectral analysis and reconstruct functional networks from electroencephalographic data to identify any neural correlates (e.g., connectivity patterns, graph-theoretical variables) of cognitive recovery after the perturbation of general anesthesia. To accomplish these objectives, we will enroll a total of 60 consenting adults aged 20–40 across the three participating sites. Half of the study subjects will receive general anesthesia slowly titrated to loss of consciousness (LOC) with an intravenous infusion of propofol and thereafter be maintained for 3 h with 1.3 age adjusted minimum alveolar concentration of isoflurane, while the other half of subjects serves as awake controls to gauge effects of repeated neurobehavioral testing, spontaneous fatigue and endogenous rest-activity patterns.

## Introduction

Understanding mechanisms of human consciousness and cognition, including reconstitution after ablation, is a fundamental pursuit in 21st century science (Dehaene and Changeux, 2011). One method of exploring the complex neural underpinnings of consciousness and cognition is to characterize how they are reconstructed after a state of unconsciousness. Recovery from coma or vegetative states offers one potential means to study this, but reversal of such states is unpredictable and slow, if it even occurs at all. Conversely, sleep is a fully reversible state of unconsciousness that is common to all humans, but recovery may be too rapid to allow characterization of the detailed temporal evolution of returning consciousness and cognition. By contrast, general anesthesia provides an excellent and reproducible model of state transitions from unconsciousness to consciousness and higher cognition.

Clinically, emergence from anesthesia is often superficially assumed to occur at a discrete temporal *point* corresponding to a patient’s ability to follow a spoken command. However, it is more likely that emergence from general anesthesia is a *process* that evolves from basic phenomenal consciousness to higher executive function over a time course amenable to careful study. This hypothesis is supported by a neuroimaging study demonstrating that anesthetic emergence, defined most primitively as the ability to respond to a command, occurs with reactivation of phylogenetically ancient portions of the human brain, with limited neocortical involvement (Långsjö et al., 2012) while recovery of testing dependent upon frontoparietal function (Usui et al., 2009) may require hours to revert to baseline (Eger et al., 1997).

Intriguingly, however, evidence suggests variability in scalp electroencephalographic oscillations as patients emerge from general anesthesia in the clinical setting (Lee et al., 2011; Chander et al., 2014). Detailed trajectories of neurobehavioral recovery following emergence are lacking (Eger et al., 1997, 1998). It is unknown which cognitive functions recover early, if neurobehavioral functions return in an invariant, sequential order, and the time scale required for complete restoration of cognitive function. For example, it is generally assumed that even when the gross effects of general anesthesia appear to have dissipated, patients might nevertheless have markedly impaired judgment and memory, but there has been insufficient formal study of this recovery pattern. If there is a universal sequence of cognitive recovery, it is probable that primitive functions are reconstituted prior to higher cognitive ones. However, it is also possible that there is marked inter-individual variation in this sequence. Such questions are germane for clinical anesthesiology but also highly relevant to neuroscience.

In an experimental setting, general anesthesia can be readily induced and thus has the potential to be an excellent model for how the brain first loses, and then reconstitutes consciousness and cognition. Using such a model could help us better understand the neural correlates of human consciousness (Mashour, 2006; Alkire et al., 2008), as well as normal and abnormal conscious state transitions, including barriers to such transitions (Voss and Sleigh, 2007; Friedman et al., 2010; Joiner et al., 2013). In the clinical setting, the return of baseline cognition after surgery is of paramount importance to the more than 300 million patients each year who have major operations worldwide (Weiser et al., 2015). Thus, a study of neurocognitive recovery from anesthesia could also advance the clinical understanding and monitoring of phenomena such as delayed emergence from anesthesia, unplanned emergence from anesthesia (i.e., awareness), delirium after anesthesia, and emergence from pathologic unconscious states such as coma.

The aims of the current study will be: (1) to measure the sequence of cognitive recovery following emergence from a prolonged state of unconsciousness with serial behavioral assessments to test the hypothesis that higher executive functions reconstitute only after more primitive functions; (2) to assess correlated changes in functional brain networks that might account for the differential recovery of cognitive function; and (3) to assess inter-individual differences in both behavioral and network recovery patterns. Further aims will include an analysis of the variability in EEG network architecture corresponding to loss of consciousness (LOC) upon a slow propofol induction, whether baseline sleep architecture affects anesthetic induction or emergence sensitivity, and whether anesthetic exposure alters subsequent rest-activity rhythms.

## Materials and Methods

### Ethics and Study Design

This is a multi-center study that has been reviewed and planned in accordance with the recommendations of the Institutional Review Boards specializing in human subjects research at the University of Pennsylvania (Protocol #818401), University of Michigan, Ann Arbor (Protocol #HUM0071578), and Washington University in St. Louis (Protocol #201308073). The research protocol was approved at all three institutions. To be eligible to participate, all subjects must give written informed consent in accordance with the Declaration of Helsinki.

### Participants

A total of 60, healthy American Society of Anesthesiologists physical status classification I or II (Vacanti et al., 1970) volunteers will be enrolled in the “Reconstructing Consciousness and Cognition” study (NCT01911195). The number of participants included in this study was informed by several factors, including previous studies (Eger et al., 1997, 1998), biological plausibility (our best guesses regarding effect size and standard deviation), and safety considerations (exposing the minimum number of humans to general anesthesia in order to answer the questions of interest). The main factor that we considered in estimating our required sample size was the time difference in return of cognitive functions within the subjects receiving general anesthesia. We modeled the sample size calculation making various assumptions regarding the difference in recovery times between cognitive domains, and the standard deviations of these parameters. For differences in recovery times between cognitive domains (possible effect sizes), we considered a range between 30 min and 90 min. For standard deviations of these parameters, we considered a range between 20 min and 40 min. With relatively conservative assumptions (difference in recovery times = 30 min and standard deviation = 40 min), 30 subjects would provide >80% power with a two-sided alpha < 0.05. With relatively liberal assumptions (difference in recovery times = 90 min and standard deviation = 20 min), 30 subjects would provide >99% power with a two-sided alpha < 0.001.

Each of the three study sites (University of Pennsylvania, University of Michigan, Washington University in St. Louis) will recruit twenty volunteers who meet the criteria displayed in Table 1. Prospective volunteers will be screened via a phone questionnaire administered by a study coordinator. Eligible subjects that give consent to participate in this study will undergo a baseline familiarization round of neurocognitive testing and be given rest-activity monitoring devices (actiwatch) 1 week prior to the study day.

**Table 1:** **Eligibility criteria for participation**.

Inclusion	Exclusion
Healthy non-obese (BMI < 30 kg/m^2^) male or female Satisfy criteria for the American Society of Anesthesiologists Physical status I or II Easily visualized uvula Ability to understand and sign informed consent documents	Age less than 20 or more than 40 years Physical indication of a difficult airway Family history of problems with anesthesia History of obstructive sleep apnea, neuropsychiatric disorders, hypertension, cardiovascular disease, reflux, sleep disorders, postoperative nausea or vomiting, or motion sickness Past or current use of psychotropic medications Current tobacco or alcohol use exceeding 2 drinks/day Reactive airway disease Positive urine toxicology test Allergy to eggs, egg products, or soy Pregnancy

### Actigraphy

In order to assess and control for differences in baseline sleep-wake rhythms and to potentially evaluate the effect of isoflurane anesthesia on subsequent rest activity behavior, subjects will be trained and instructed to wear a wrist actiwatch (ActiGraph wActisleep+) continuously at their baseline visit at least 1 week before their assigned study day. Following the completion of the study day, subjects will wear the actiwatch until the device is collected 1 week after completing the study. Raw activity counts for each subject will be collected in 1-min epochs and analyzed according to published protocols (Cole et al., 1992).

### Study Day

Subjects will be randomly assigned to receive anesthesia or to engage in restful activity on the study day as a control participant.

#### Anesthesia Volunteer Group

On the day of the study, healthy volunteers (*n* = 30) must submit a urine sample for repeat urine toxicology and, if applicable, a urine pregnancy test. Two attending anesthesiologists will be present during the study day to conduct a standard clinical preoperative history and exam, independently verify that the volunteer meets inclusion criteria and has fasted for 8 h prior to their arrival, and safely conduct the anesthetic.

Each subject will receive an intravenous catheter. An appropriately fitted EEG head cap (Electrical Geodesics, Inc. Eugene, OR, USA) will be affixed to the scalp. Electrical impedances on each channel will be kept under 50 kOhms/channel whenever possible. Standard anesthesia monitors (electrocardiogram, non-invasive blood pressure cuff, a pulse oximeter) will be applied, and capnography will be measured. Subjects will next complete a second baseline round of neurocognitive testing with ongoing EEG recordings. Upon completion of the neurocognitive battery, the subjects will be pre-oxygenated by face mask prior to induction of general anesthesia with a stepwise increasing infusion rate of propofol: 100 mcg/kg/min × 5 min increasing to 200 mcg/kg/min × 5 min, and then to 300 mcg/kg/min × 5 min. During this time, an audio loop command will be issued every 30 s to ask the subject to randomly squeeze their left or right hand twice. LOC will be recorded as the first time that a subject fails to respond to two consecutive commands. After 15 min of propofol administration, subjects will begin inhaling 1.3 age adjusted MAC of isoflurane (Nickalls and Mapleson, 2003). Thereafter, a laryngeal mask will be inserted orally, a nasopharyngeal temperature probe will be placed, and the propofol infusion will be discontinued. Anesthetized subjects will receive 1.3 age-adjusted MAC inhaled isoflurane anesthesia for 3 h. Blood pressure will be kept within 20% of baseline pre-induction values using a phenylephrine infusion or intermittent boluses of ephedrine, as necessary. Subjects will be placed on pressure support ventilation with pressures titrated to maintain tidal volumes in the 5–8 ml/kg range and end tidal carbon dioxide levels between 35–45 torr. Surface warming blankets will be applied to maintain body temperature in the normal range. For antiemetic prophylaxis, subjects will receive intravenous ondansetron 4 mg 30 min prior to discontinuation of isoflurane.

At the end of the 3-h anesthetic period, the isoflurane will be discontinued and the verbal command loops will be reissued every 30 s. The laryngeal mask will be removed when deemed medically safe by the attending anesthesiologists. Recovery of consciousness (ROC) will be defined as the earliest instance in which subjects correctly respond to two consecutive audio loop commands. At this point, defined as time = 0 min, the subject will begin taking the neurocognitive battery of tests followed by a brief resting period. Neurocognitive testing is to be repeated at *t* = 30, 60, 90, 120, 150 and 180 min following emergence. Each battery of neurocognitive testing will last approximately 15–25 min and will be preceded by 5 min of eyes closed, resting state EEG data acquisition. Subjects will be permitted a brief break to use the restroom and eat or drink, as necessary.

Subjects will be discharged according to standard post-anesthesia care unit discharge criteria after completing their final battery of neurocognitive testing. A study site coordinator will contact each subject within 24 h of the study day to document any adverse events.

#### Control Group

A second group of healthy individuals (*n* = 30) will be recruited to participate in the same study design. These individuals are also fasted overnight, but will not have intravenous lines inserted. Instead of being anesthetized, these volunteers will remain awake (by reading or watching television on a personal electronic device) and continue fasting for 3.5 h and serve as controls both for potential learning effects of repeated testing and also for circadian variability in testing performance (McLeod et al., 1982; Gur et al., 2001; Van Dongen et al., 2003; Van Dongen and Dinges, 2005; Jasper et al., 2009; Tucker et al., 2010). Importantly, subjects randomized to the restful group will be instructed to avoid napping and will be regularly monitored by a dedicated research assistant.

### EEG

In order to assess neural correlates of the anesthetic state as well as the return of cognitive functions after general anesthesia, each participant enrolled at the University of Pennsylvania and University of Washington in St. Louis (*n* = 40) will be fitted with 32 EEG scalp electrodes. Subjects at the University of Michigan (*n* = 20) will be fitted with higher density 128 EEG scalp electrodes. EEG recordings will begin prior to the baseline neurocognitive testing on the study day and will be acquired on a near-continuous basis until the completion of the final neurocognitive test. We will conduct time- and frequency-domain analysis of the EEG, and also apply techniques to measure functional connectivity and graph-theoretical variables as follows.

#### Spectral Analysis

Chronux’s^1^ multitaper method will be used to compute spectrograms (Mitra and Bokil, 2008) with the following parameters: window length *T* = 2 s, step size = 0.1 s, time-bandwidth product *NW* = 2, and number of tapers *K* = 3.

#### Source Estimation

A distributed model consisting of 10,000 current dipoles will be used for mapping cortical current source density. A standard Montreal Neurological Institute brain model will be employed for constraining dipole locations and orientations. The Brainstorm software package (Tadel et al., 2011) will subsequently be used to warp dipole locations and orientations to the geometry of the EEG sensor net.

#### Functional Connectivity

Phase Lag Index (PLI), a measure that addresses the problem of volume conduction by accounting only for non-zero phase lead/lag relationships (Stam et al., 2007), will be used to assess functional connectivity.

#### Graph Theory Analysis

After producing a binary adjacency matrix, basic network topological properties, including average path length, clustering coefficient, modularity and global efficiency will be calculated. The average path length (L_w_) is defined as the average of the shortest path lengths (L_ij_) between all pairs of nodes in the network (Latora and Marchiori, 2001). The clustering coefficient indicates how graph nodes organize. Higher values signify networks with highly clustered or regular structures (Watts and Strogatz, 1998). The clustering coefficient (C_w_) for the network is calculated by averaging the clustering coefficients of all individual nodes (C_i_). The network modularity corresponds to the sum of connection strengths within modules and will be calculated using the Louvain algorithm in the brain connectivity toolbox (Rubinov and Sporns, 2010). High modularity values indicate networks with strong within-module connections and weak between-module connections (Newman, 2006). Finally, the global efficiency is defined as the inverse of the average shortest path length over all pairs of nodes. All network metrics will be normalized against randomized networks.

### Neurocognitive Testing

Neurocognitive tests have been selected from the Cognition test battery (Basner et al., 2015) to reflect a broad range of cognitive domains, ranging from basic abilities such as sensory-motor speed to complex executive functions such as abstraction. On the study day, subjects will complete all neurocognitive testing while sitting upright in an operating room hospital bed using a laptop computer calibrated for timing accuracy. The order of the six tests will be randomized but balanced across subjects. Each individual subject will always receive the tests in the same order (except during familiarization). In each test session, subjects will repeat the first test after completion of test #6. Therefore, the temporal resolution for one test in five control and five experimental subjects will be doubled. The following six tests, adopted from Basner et al. (2015), were chosen for this study.

#### Motor Praxis

The Motor Praxis Task (MP) measures sensorimotor speed and also ensures that volunteers have sufficient command of the computer interface. Volunteers are instructed to click on squares that randomly appear on the monitor. Successive squares are increasingly smaller and more difficult to track than preceding ones. Performance is determined by the duration of time required for participants to click within each of the 20 consecutive squares. The test primarily recruits and depends upon function of sensorimotor cortex (Gur et al., 2001, 2010; Neves et al., 2014).

#### Psychomotor Vigilance Test

The Psychomotor Vigilance Test (PVT) measures a volunteer’s reaction times (RT) to visual stimuli that are presented at random inter-stimulus intervals. Volunteers are instructed to monitor a box on the screen, and depress the space bar as soon as a millisecond clock appears and begins timing response latency. Subjects’ RT will be displayed for 1 s. Volunteers are instructed to respond as quickly as possible without depressing the spacebar in the absence of a stimulus. Such erroneous responses are considered false starts (errors of commission). The PVT measures vigilant attention and is extremely sensitive to the effects of both acute and chronic sleep deprivation as well as circadian misalignment. Importantly, repeated psychomotor vigilance testing does not improve a volunteer’s performance as it is not confounded by learning effects (Basner and Dinges, 2011). In the well-rested state, or whenever sustained attention performance is optimal, right frontoparietal cortical regions exhibit activation. Conversely, following sleep deprivation and suboptimal performance studies reveal increased activation of default-mode networks, which is considered to be a compensatory mechanism (Drummond et al., 2005). The Cognition software given to our volunteers will use a previously validated, 3-min version of the PVT with a 2–5 s inter-stimulus interval (Basner et al., 2011).

#### Digit Symbol Substitution Test

The Digit-Symbol Substitution Task (DSST) adapts the Wechsler Adult Intelligence Scale (WAIS-III) for a computerized presentation. The DSST measures cognitive throughput and requires volunteers to refer to a displayed legend to decode specific symbols with each of the digits one through nine. During testing, a symbol appears on the computer screen. Volunteers must select the corresponding number as quickly as possible. Volunteers have a total of 90 s to decode as many symbols as possible. The digit-symbol translation key is randomly re-assigned with each test administration. The DSST primarily recruits the temporal cortex, prefrontal cortex and motor cortex. Activation of frontoparietal cortices during DSST performance has been interpreted as reflecting both onboard processing in working memory and low-level visual search (Usui et al., 2009).

#### Fractal-2-Back

The Fractal 2-Back (F2B) is a more challenging nonverbal variant of the Letter 2-Back test. N-back tests probe working memory. The F2B consists of the sequential 750 ms display of 62 fractal figures. Each fractal image may be displayed multiple times during the presentation. For correct performance, volunteers must respond when the current stimulus matches that displayed two figures ago. The F2B is well-validated and known to robustly activate dorsolateral prefrontal cortex, cingulate and hippocampus (Ragland et al., 2002).

#### Visual Object Learning

The Visual Object Learning Test (VOLT) measures the volunteer’s memory for complex figures (Glahn et al., 1997). Volunteers are asked to memorize 10 three-dimensional figures that are sequentially each displayed for 5 s. Next, volunteers are instructed to distinguish the 10 memorized objects from a larger set of 20. The testing set of 20 includes images from the learning set as well as similar yet novel ones. Visual object learning tasks have been shown to depend upon frontal and bilateral anterior medial temporal cortices as well as the hippocampus (Jackson and Schacter, 2004).

#### Abstract Matching

The Abstract Matching (AM) test (Glahn et al., 2000) is a validated test of executive function that depends upon abstraction and concept formation. This task requires volunteers to discern general rules from discrete examples. Volunteers are shown two pairs of objects at the bottom left and right of a computer screen. The objects’ properties are varied by perceptual dimensions (e.g., shape and color). Volunteers are shown a target object that must be classified as belonging to one of the two pairs, based on a set of implicit, abstracted rules. This test uses 30 rounds of object classification. Tasks such as the AM paradigm evaluate abstraction and concept formation depending upon the prefrontal cortex (Berman et al., 1995).

## Discussion

This study has been designed to evaluate the neurobehavioral and brain network recovery as healthy humans emerge from a deep state of general anesthesia. Remarkably, despite more than 170 years of continuous use in humans, fundamental questions about how central nervous system functions are re-established following exit from the anesthetic state remain unanswered. Although cognitive assessments do indeed occur as part of routine practice, typical clinical evaluations merely gage when patients first regain the ability to follow commands. An initial study of emergence from anesthesia in surgical patients using frontal EEG channels suggests discrete clusters of functional heterogeneity in electroencephalographic rhythms as patients regained consciousness (Chander et al., 2014). However, unambiguous assignment of spectral patterns in the complex setting of clinical polypharmacy and varying surgical insults remains challenging. An observational cohort study of more than 700 patients suggested that EEG activity patterns during the maintenance phase could predict postoperative recovery as those who experienced intraoperative EEG suppression who were more likely to exhibit delirium (Fritz et al., 2016). Several clinical studies have indeed coarsely assessed neurobehavioral recovery using mini-mental status exams, Trieger dot, P-deletion tests, or digit symbol substitution tests, but these studies have been primarily designed to assess the speed of recovery by comparing groups of patients exposed to distinct anesthetic drugs (Tsai et al., 1992; Eger et al., 1997, 1998; Chen et al., 2001). Detailed, serial evaluations of an individual’s cognitive capacity upon emergence, and the variability of the recovery sequence are still lacking. Moreover, as there is ongoing learning that occurs during serial testing, the inclusion of a non-anesthetized control cohort in this study is deemed to be an essential methodological component. Neurobehavioral studies are critical to determine the sequence of events that reveal a normal pattern of anesthetic emergence in contrast to pathologic emergence that may denote the onset of delirium. More generally though, detailed investigation of anesthetic emergence offers a window into the evolution of higher human cognition.

Based upon previous studies, we planned to deliver an isoflurane anesthetic at 1.3 age adjusted minimum alveolar concentration for 3 h as this anesthetic has minimal adverse hemodynamic consequences in healthy volunteers (Eger et al., 1997). Hence, our cumulative anesthetic dose will approach four MAC-hours and will nicely approximate actual clinical conditions. The choice of studying healthy volunteers was made to define the normal sequence of events in a setting devoid of surgical or inflammatory insults, disease heterogeneity and ensuing complexity, or polypharmacy. To maximize the likelihood of determining whether emergence occurs simultaneously independent of an individual cognitive domain or whether there would be an ordered and invariant sequence akin to rebooting a computer, we chose the volatile anesthetic isoflurane, based upon its blood gas solubility, which exceeds that of both desflurane and sevoflurane (Soares et al., 2012). We reasoned that should a stereotyped pattern exist, the temporal splaying of isoflurane emergence would optimize our ability to capture distinct rate constants for the return of memory, abstract reasoning, attention, sensorimotor fluidity and cognitive processing speed as measured by the Cognition test battery. Since each round of Cognition testing captures all of the aforementioned domains and requires a total of 15–25 min to complete, we planned serial testing across emergence every 30 min. Double density testing of one cognitive domain in each of the subjects roughly every 15 min is hoped to provide finer temporal resolution of emergence while still enabling a workflow in volunteers that is not too onerous.

Our experimental design also included the capture of continuous EEG with montages varying from 32–128 channels. Recordings taken at baseline during cognition testing and across the subsequent serial evaluations that follow anesthetic emergence may also reveal EEG network signatures of performance that can be correlated with neurobehavioral recovery. Moreover, this study will also permit rigorous evaluation of several markers of loss and return of consciousness including those based upon graph theory and dynamic criticality (Jordan et al., 2013; Lee et al., 2013; Purdon et al., 2013; Akeju et al., 2014; Chennu et al., 2014; Untergehrer et al., 2014; Solovey et al., 2015; Ranft et al., 2016).

Given the well established sensitivity of cognition testing to sleep deprivation and fatigue (Basner et al., 2011, 2015), a critical quality metric for this experiment will be to ensure that subjects are well rested prior to neurobehavioral testing and that potential differences in intrinsic rest-activity patterns are known. One added benefit of measuring actigraphy after the test day is the potential to evaluate post-anesthesia effects on rest-activity rhythms. There is increasing recognition that anesthetics themselves have the potential to affect neural circuits regulating sleep and wakefulness (Alkire et al., 2008; Franks, 2008; Moore et al., 2012). Anesthetic exposure has the potential to phase shift circadian clocks and older reports also suggest that anesthetics may disrupt sleep wake rhythms (Dispersyn et al., 2009). Our study will also utilize wrist actigraphy to evaluate subsequent sleep in humans (Cole et al., 1992) and directly determine if a 4-MAC hour isoflurane exposure grossly alters sleep architecture, which is disrupted following surgery (Moote and Knill, 1988; Knill et al., 1990).

The evolution of cognition at and following emergence from anesthesia remains poorly understood. The knowledge gained from this study has the potential to provide insight into the neurobiological mechanisms that underlie emergence from anesthesia as well as to contribute towards the development of diagnostic tools that can be used to screen for emergence abnormalities.

## Author Contributions

MSA, MBK, GAM conceived and designed the study; KLM, ARM-W, BJAP, VT, SB-M, MB helped to prepare and test equipment critical for the preparation and conduct of the protocol; KLM and MBK wrote the manuscript; all authors contributed to critical review of the manuscript.

## Conflict of Interest Statement

The authors declare that the research was conducted in the absence of any commercial or financial relationships that could be construed as a potential conflict of interest.

## Footnotes

^1^www.chronux.org
